# Investigating Clinicians’ Intentions and Influencing Factors for Using an Intelligence-Enabled Diagnostic Clinical Decision Support System in Health Care Systems: Cross-Sectional Survey

**DOI:** 10.2196/62732

**Published:** 2025-04-07

**Authors:** Rui Zheng, Xiao Jiang, Li Shen, Tianrui He, Mengting Ji, Xingyi Li, Guangjun Yu

**Affiliations:** 1 Shanghai Children's Hospital Shanghai China; 2 School of Public Health School of Medicine Shanghai Jiao Tong University Shanghai China; 3 Clinical Research Center Shanghai Sixth People's Hospital Affiliated to Shanghai Jiao Tong University School of Medicine Shanghai China; 4 Zhongshan Hospital Institute of Clinical Science Fudan University Shanghai Medical College Shanghai China; 5 Renji Hospital School of Medicine Shanghai Jiao Tong University Shanghai China; 6 Shanghai Chest Hospital School of Medicine Shanghai Jiao Tong University Shanghai China

**Keywords:** artificial intelligence, clinical decision support systems, task-technology fit, technology acceptance model, perceived risk, performance expectations, intention to use

## Abstract

**Background:**

An intelligence-enabled clinical decision support system (CDSS) is a computerized system that integrates medical knowledge, patient data, and clinical guidelines to assist health care providers make clinical decisions. Research studies have shown that CDSS utilization rates have not met expectations. Clinicians’ intentions and their attitudes determine the use and promotion of CDSS in clinical practice.

**Objective:**

The aim of this study was to enhance the successful utilization of CDSS by analyzing the pivotal factors that influence clinicians’ intentions to adopt it and by putting forward targeted management recommendations.

**Methods:**

This study proposed a research model grounded in the task-technology fit model and the technology acceptance model, which was then tested through a cross-sectional survey. The measurement instrument comprised demographic characteristics, multi-item scales, and an open-ended query regarding areas where clinicians perceived the system required improvement. We leveraged structural equation modeling to assess the direct and indirect effects of “task-technology fit” and “perceived ease of use” on clinicians’ intentions to use the CDSS when mediated by “performance expectation” and “perceived risk.” We collated and analyzed the responses to the open-ended question.

**Results:**

We collected a total of 247 questionnaires. The model explained 65.8% of the variance in use intention. Performance expectations (β=0.228; *P*<.001) and perceived risk (β=–0.579; *P*<.001) were both significant predictors of use intention. Task-technology fit (β=–0.281; *P*<.001) and perceived ease of use (β=–0.377; *P*<.001) negatively affected perceived risk. Perceived risk (β=–0.308; *P*<.001) negatively affected performance expectations. Task-technology fit positively affected perceived ease of use (β=0.692; *P*<.001) and performance expectations (β=0.508; *P*<.001). Task characteristics (β=0.168; *P*<.001) and technology characteristics (β=0.749; *P*<.001) positively affected task-technology fit. Contrary to expectations, perceived ease of use (β=0.108; *P*=.07) did not have a significant impact on use intention. From the open-ended question, 3 main themes emerged regarding clinicians’ perceived deficiencies in CDSS: system security risks, personalized interaction, seamless integration.

**Conclusions:**

Perceived risk and performance expectations were direct determinants of clinicians’ adoption of CDSS, significantly influenced by task-technology fit and perceived ease of use. In the future, increasing transparency within CDSS and fostering trust between clinicians and technology should be prioritized. Furthermore, focusing on personalized interactions and ensuring seamless integration into clinical workflows are crucial steps moving forward.

## Introduction

### Background

The complexity of modern medical information and the rapid updating of medical knowledge make it difficult for clinical staff to master the latest diagnostic and treatment information. Medical errors are an important cause of a poor prognosis in patients. At the same time, high-quality medical resources are often concentrated in big cities and large-sized medical institutions, while those in grassroots and remote areas are relatively scarce. Therefore, how to improve the efficiency and accuracy of clinical diagnosis and treatment is an important clinical and public health issue at present [1].

In recent years, with the rapid development of artificial intelligence (AI) technology, the integration and practical application of AI in health care have provided a promising solution to address the aforementioned issues. AI-enabled clinical decision support systems (CDSS) have become a core concept for leveraging technology to support the health care field [2]. CDSS are the results of combining traditional decision support systems and AI. CDSS are designed to enhance medical decision-making by using targeted clinical knowledge, patient information, and other health data to improve health care services. CDSS are typically implemented as web applications or integrated with electronic health records and computerized physician order entry systems. For intelligent diagnostic assistance, CDSS use natural language processing techniques to analyze unstructured text data such as patient complaints, medical histories, and physical signs. They automatically extract key clinical features, dynamically matching them with disease-symptom associations in knowledge graphs. CDSS also incorporate evidence-based medical rules and updated clinical guidelines to emulate expert reasoning and assess potential causes, complications, and rare disease risks. Through interactive visualization interfaces, they present diagnostic rationales, risk alerts, and recommended diagnostic pathways, ensuring decision logic remains fully traceable [3].

Previous studies have suggested that CDSS hold promise for enhancing clinician performance, promoting patient safety, and improving the overall quality of health care [4-7]. However, the potential of CDSS in medicine remains underutilized [8]. Various tertiary hospitals in Shanghai, as leaders in digital transformation, are starting to implement CDSS. Despite this, hospital investment in CDSS does not always achieve the desired results, and there may even be a negative correlation between inputs and outputs [9]. A CDSS was deployed in a tertiary hospital in Shanghai for 12 months, resulting in a lower-than-expected utilization rate (43% for the tertiary hospital) due to clinicians’ distrust of the system and its poor integration into clinicians’ workflows [2]. The perceptions of clinicians, as end users of the system, will ultimately influence the development of a CDSS [10]. Therefore, it is crucial to understand the key factors that influence clinicians’ intent to use a CDSS, yet limited studies are available [11].

Therefore, we had 3 aims for this study. First, we attempted to identify the factors influencing clinician intent to use a CDSS via a theoretical model. Second, we empirically examined the applicability of the model in the context of implementing a CDSS. Third, we proposed corresponding managerial implications based on the results.

### Theory and Related Work

A number of studies exploring the use of CDSS from the perspective of human factors have applied a variety of theoretical models, including but not limited to the technology acceptance model (TAM) [12-14], stating that clinicians’ interactions with CDSS are influenced by their overarching perceptions of technology. These perceptions encompass their attitudes, beliefs, and experiences with various technological tools and systems, which collectively shape their acceptance and utilization of CDSS. TAM elucidates how perceived ease of use and perceived usefulness act as intermediary factors between system characteristics and its utilization [11].

There are additional studies that consider the specificity of information technology in the health care field and use the task-technology fit (TTF) framework to assess the level of support provided by information technology to clinicians’ work [15,16]. The TTF framework evaluates how well the characteristics of technology and the requirements of tasks align to enhance user performance. By analyzing both technology and task characteristics, the model aims to identify areas where adjustments or improvements can be made to better meet user needs and optimize performance [17]. The TTF framework has undergone empirical validation across diverse settings, encompassing health care domains such as hospital information systems and electronic health records [15,18].

Each model exhibits unique advantages. TAM primarily focuses on exploring user behaviors and trends, emphasizing users’ perceptions of the technology’s ease of use and perceived usefulness. Nevertheless, TAM may not comprehensively take into account specific task requirements. Conversely, the TTF model heavily emphasizes assessing the congruence between technology and task characteristics, focusing on how well the technology aligns with the task’s demands. It offers valuable insights into how effectively the technology facilitates the efficient, effective accomplishment of tasks.

Several studies have integrated the TTF model with TAM, demonstrating synergistic effects between the two models. This integration highlights the importance of both user perceptions and task-technology alignment, thus providing a more comprehensive understanding of user behavior and system effectiveness than either model alone [19-22]. By integrating TAM and the TTF model, researchers can harness the strengths of both, offering a more comprehensive understanding of user acceptance and system performance. Previous research has substantiated the substantial influence of the task-technology fit on perceived ease of use. This validation underscores the critical role of aligning technology with task requirements in shaping users’ perceptions of how easy the system is to use and how beneficial it is for their tasks [23]. Therefore, the TTF model can serve as a precursor factor influencing perceived ease of use. Based on this rationale, this study selected the core variable of “perceived ease of use” from the TAM.

Given the complexity and constant evolution of AI, it has yet to become a cornerstone of the health care system or medical education. The lingering uncertainty regarding the safety and potential risks posed by AI to patients remains a pivotal factor influencing clinicians’ intentions to adopt the technology [11,24,25]. At the same time, the significant impact of task-technology fit and perceived ease of use on perceived risk has also been verified [26,27]. Therefore, this study further incorporated the variable of “perceived risk” into the research framework., aiming to deepen the comprehension of clinicians’ tendencies to adopt a CDSS.

**Figure 1:**
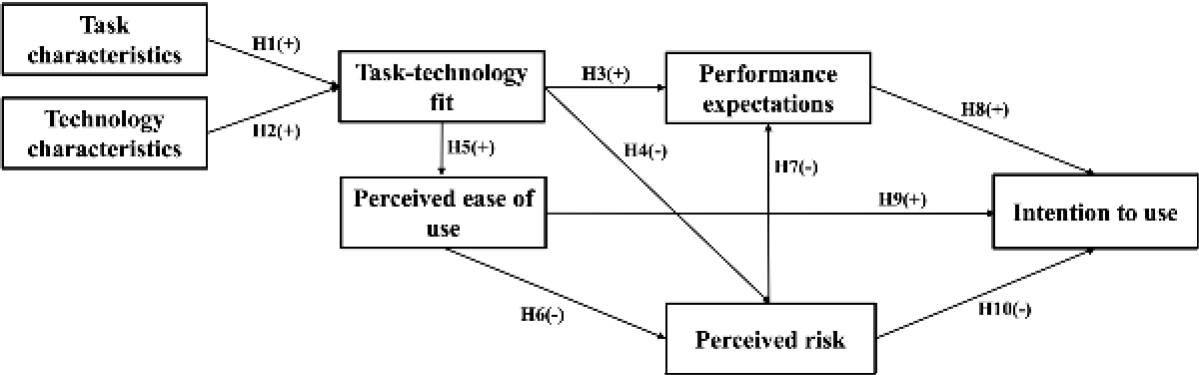
Conceptual model. +: positive effect; -: negative effect; H: hypothesis.

Drawing upon the theoretical underpinnings and existing research findings, we herein introduced a theoretical model (Figure 1) along with the corresponding hypotheses: (1) task characteristics positively affect the task-technology fit (hypothesis 1), (2) technology characteristics positively affect the task-technology fit (hypothesis 2), (3) task-technology fit positively affects performance expectations (hypothesis 3), (4) task-technology fit negatively affects perceived risk (hypothesis 4), (5) task-technology fit positively affects perceived ease of use (hypothesis 5), (6) perceived ease of use negatively affects perceived risk (hypothesis 6), (7) perceived risk negatively affects performance expectations (hypothesis 7), (8) performance expectations positively affects intention to use (hypothesis 8), (9) perceived ease of use positively affects intention to use (hypothesis 9), and (10) perceived risk negatively affects negatively affect intention to use (hypothesis 10).

## Methods

### Study Design and Setting

We conducted the study in 3 tertiary hospitals (Shanghai Children’s Hospital, Ren Ji Hospital, and Shanghai Sixth People’s Hospital) in Shanghai. The study involved administering a questionnaire survey to 247 clinicians across the inpatient and outpatient departments of the 3 hospitals. The study spanned a duration of 4 months, from December 2023 to March 2024.

### Sample Size and Sampling

Marcoulides and Saunders [28] contended that the minimum sample size is contingent on the maximum number of arrows directed toward the latent variable. Although establishing a suitable sample size is crucial for structural equation modeling (SEM), consensus on the ideal sample size within the literature is lacking. Evidence suggests that even simple SEM can yield meaningful results with small sample sizes. However, as a general guideline, a minimum sample size of 100 to 150 is often recommended for conducting SEM analyses [29]. Simple random sampling was used for this study, with a total of 247 clinicians participating in and completing the study. This sample size is sufficient to yield statistically significant results.

### Measurement Instruments

The questionnaire comprised 3 sections: demographic characteristics, multiple-item scales, and an optional open-ended question (“What deficiencies do you identify in the CDSS?”). The 7 constructs within the model were evaluated using multi-item scales adapted from those by Davis [30], Stone and Grønhaug [31], Goodhue [32], and Venkatesh et al [33], with modifications made to the original items to align with the context of this research, which primarily focuses on clinicians’ attitudes toward CDSS use. The items were scored using 5-point Likert scales. Table 1 presents the origins and definitions of the constructs.

**Table 1:** Definitions of the constructs.

Construct	Operational definition	Reference
Task characteristics	Those that might move a user to rely more heavily on certain aspects of the information	Goodhue and Thompson [34]
Technology characteristics	The characteristics of using a CDSS^a^ during clinicians’ operation of it	Goodhue and Thompson [34]
Task-technology fit	The degree to which a clinician believes that using a CDSS would enhance his or her job performance	Stone and Grønhaug [31]
Performance expectations	The performance-related consequence of the behavior, specifically performance expectations that deal with job-related outcomes	Venkatesh et al [33]
Perceived ease of use	The degree to which clinicians believe that using a CDSS would be free from effort	Davis [30]
Perceived risk	An assumption of risk on the part of clinicians with the use of a CDSS	Stone and Grønhaug [31]
Intention to use	Clinicians’ intention to use a CDSS	Venkatesh et al [33]

^a^CDSS: clinical decision support system.

Multimedia Appendix 1 contains the items corresponding to each construct along with their respective sources. We conducted a pretest involving 63 clinicians, and the results indicated that the questionnaire demonstrated good reliability and validity.

### Data Collection and Recruitment

We specifically included clinicians with varying levels of seniority and educational backgrounds. We distributed an email to clinicians affiliated with the hospital through list servers. The email outlined the objectives of the study, gave an overview of the CDSS, and contained a link to the online survey. Interested clinicians voluntarily participated after providing their consent. A total of 247 clinicians participated in the study, and all 247 questionnaires collected were audited and considered valid. In addition, we randomly selected 48 clinicians and administered an open-ended survey to garner their insights on the shortcomings and potential improvements of the CDSS.

### Statistical Analysis

Frequencies and percentages were used to describe the characteristics of the clinicians. Analyses were carried out using SPSS version 25.0 (IBM Corp). After analyzing the descriptive statistics, the next step in the research process was to validate the model and test the hypotheses using a partial least squares SEM analysis. This analysis was conducted in Smart PLS4. Partial least squares SEM is a variance-based approach that does not assume multivariate normality, making it robust for analyzing data with non-normal distributions and small sample sizes.

The implementation of the method involves a 2-step process [35]. The first step involves using the partial least squares algorithm to evaluate the reliability and validity of the measurement model. In the second step, we assessed the fit of the structural model and tested hypotheses using bootstrapping.

The open-ended question was analyzed via thematic analysis, analyzing the number of themes and the frequency of occurrence of each theme.

### Ethics Approval

This study was approved by the Ethics Committee of Shanghai Children's Hospital (approval number: 2021R077-E01). All clinical physicians participated in this study voluntarily, without compensation, and anonymously under informed consent. They retained the right to decline to answer any survey questions or withdraw from the study at any time. All collected data were thoroughly de-identified to ensure participant confidentiality

## Results

### Demographic Information

The study involved the participation of 247 clinicians, and all valid questionnaires were collected. In Table 2, the demographic information of the clinicians is presented. A total of 129 (129/247, 52.2%) men and 118 (118/247, 47.8%) women participated in the study, with ages ranging between 25 years and 55 years. Individuals aged 25 years to 40 years constituted the majority, comprising 62.8% (155/247) of the participants. Regarding professional titles, resident physicians (66/247, 26.7%) and attending physicians (60/247, 24.3%) were the predominant groups.

**Table 2:** Participant characteristics (N=247).

Participant characteristics			Participants, n (%)
**Gender**			
	Men	129 (52.2)	
	Women	118 (47.8)	
**Age (years)**			
	≤24	47 (19)	
	25-40	155 (62.8)	
	≥41	45 (18.2)	
**Professional position**			
	Resident physician	66 (26.7)	
	Attending physician	60 (24.3)	
	Deputy chief physician	38 (15.4)	
	Chief physician	8 (3.2)	
	Others	75 (30.4)	
**Working experience (years)**			
	<1	40 (16.2)	
	1-10	102 (41.3)	
	11-20	71 (28.7)	
	≥21	34 (13.8)	
**System usage time (years)**			
	<1	106 (42.8)	
	1-3	56 (22.7)	
	4-5	32 (13)	
	≥6	53 (21.5)	

### Intention to Use a CDSS Dimensional Scores

The average scores of the dimension items in this study were as follows: clinicians' task characteristics (4.45, SD 0.87), technological characteristics (3.97, SD 0.80), task-technology fit (4.20, SD 0.74), performance expectancy (4.14, SD 0.78), perceived ease of use (4.03, SD 0.92), perceived risk (1.80, SD 0.85), and intention to use (3.88, SD 1.28).

### Measurement Model Assessment

We typically assessed the reliability of each latent construct (eg, factors, variables) using measures like composite reliability or Cronbach α [36]. Furthermore, we assessed the convergent validity by examining the loadings of the indicators on their respective constructs and the average variance extracted (AVE) [37]. The outcomes of this analysis are summarized in Table 3. The results presented in Table 3 reveal that all Cronbach α and composite reliability values exceeded 0.7, indicating solid internal consistency and reliability for each construct. Moreover, the AVE for each construct surpassed 0.5, signifying adequate convergent validity. Additionally, the factor loadings for each item were above 0.7, suggesting that each item reliably measures its respective construct. Collectively, these findings demonstrate robust reliability and convergent validity for the measurement model.

**Table 3:** Construct reliability and convergent validity.

Constructs and items	CR^a^	AVE^b^	Cronbach α	Factor loading
TAC^c^	0.965	0.933	0.930	—^d^
TAC1	—	—	—	0.974
TAC2	—	—	—	0.958
TEC^e^	0.961	0.924	0.918	—
TEC1	—	—	—	0.963
TEC2	—	—	—	0.960
TTF^f^	0.939	0.886	0.871	—
TTF1	—	—	—	0.947
TTF2	—	—	—	0.935
PEOU^g^	0.940	0.797	0.915	—
PEOU1	—	—	—	0.890
PEOU2	—	—	—	0.854
PEOU3	—	—	—	0.936
PEOU4	—	—	—	0.890
PE^h^	0.928	0.812	0.884	—
PE1	—	—	—	0.913
PE2	—	—	—	0.915
PE3	—	—	—	0.875
PR^i^	0.939	0.836	0.902	—
PR1	—	—	—	0.888
PR2	—	—	—	0.915
PR3	—	—	—	0.939
ITU^j^	0.960	0.923	0.916	—
ITU1	—	—	—	0.961
ITU2	—	—	—	0.961

^a^CR: composite score.

^b^AVE: average variance extracted.

^c^TAC: task characteristics.

^d^Not applicable.

^e^TEC: technology characteristics.

^f^TTF: task-technology fit.

^g^PEOU: perceived ease of use.

^h^PE: performance expectations.

^i^PR: perceived risk.

^j^ITU: intention to use.

Moreover, discriminant validity was evaluated to ensure that the constructs in the measurement model were distinct from each other. Discriminant validity is a concept in research and statistics that assesses the extent to which different measures or constructs truly represent distinct concepts or variables. This analysis helped confirm that the measures intended to represent different constructs do not overlap substantially. By examining the correlations between constructs and comparing them with the square root of the AVE for each construct, we could determine whether the measures exhibit adequate discriminant validity. The outcomes of this analysis are summarized in Table 4. As evidenced in Table 4, the outcomes confirm that discriminant validity was achieved. This is evident by ensuring that the square root of the AVE for each construct exceeded the correlations between that construct and other constructs. Importantly, this criterion was met for all constructs included in the analysis. Consequently, the measurement model successfully demonstrated discriminant validity, indicating that the constructs are distinct from each other as intended [37] based on the comprehensive assessment of the measurement model, which included evaluating construct reliability, convergent validity, and discriminant validity. As a result, we could proceed with confidence to test the research hypotheses using this robust measurement model.

**Table 4:** Discriminant validity.

Constructs	Task characteristics	Technology characteristics	Task-technology fit	Performance expectations	Perceived ease of use	Perceived risk	Intention to use
Task characteristics	0.966	0.103	0.245	0.084	0.076	–0.054	0.039
Technology characteristics	0.103	0.962	0.767	0.678	0.667	–0.394	–0.542
Task-technology fit	0.245	0.767	0.941	0.901	0.689	–0.583	0.640
Performance expectations	0.084	0.678	0.675	0.901	0.893	–0.571	0.595
Perceived ease of use	0.076	0.667	0.692	0.689	0.893	0.914	–0.774
Perceived risk	–0.054	–0.394	–0.542	–0.583	–0.571	0.914	0.961
Intention to use	0.039	0.370	0.468	0.640	0.595	–0.774	0.961

### Structure Model Assessment

**Figure 2:**
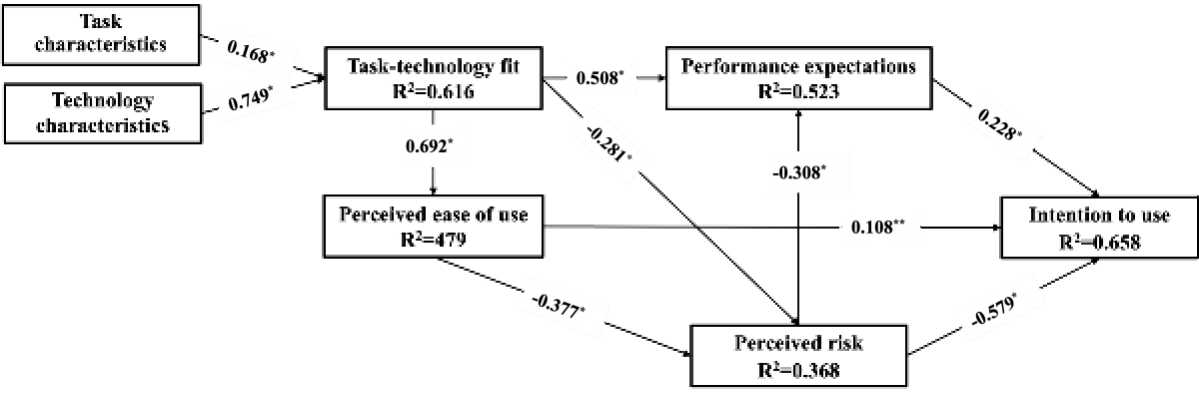
Result of the structure model. *P<.001; **P>.05.

In our research, all variance inflation factors were below the predefined cutoff value of 5. Therefore, we concluded that no multicollinearity was present in our data set [38]. The assessment of the research model involved evaluating the path coefficients (β) and coefficients of determination (R²). Table 5 presents the path coefficients along with their significance levels, hypothesis outcomes, and R²values. Additionally, Figure 2 provides a visual representation of the research model, illustrating the relationships between the variables and highlighting the significant paths identified through the analysis. These results offer insights into the strength and direction of the relationships between the variables within the model, as well as the extent to which they explain the variance in the dependent variables. The coefficient of determination, or R², represents the proportion of variance in the endogenous latent variable (in this case, “intention to use”) that is accounted for by the predictors included in the model. In our analysis, the entire model explained 65.8% of the variance in “intention to use.” This level of explained variance is considered substantial, indicating that a significant portion of the variability in the intention to use can be attributed to the predictors included in the model. The path coefficients (β) indicate the strength and direction of the direct effects of independent variables on dependent variables in the structural model. In our analysis, hypotheses based on the TTF framework (hypotheses 1, 2, 3, 8) were all supported, suggesting significant relationships between the TTF model constructs and the specified dependent variables. Similarly, hypotheses related to the newly integrated constructs, perceived ease of use and perceived risk (hypotheses 4, 5, 6, 7, 10), were also supported, indicating significant direct effects between these constructs and the specified dependent variables. However, hypothesis 9, presumably involving a relationship between one of the newly integrated constructs and a dependent variable, was not supported by the data.

**Table 5:** Hypothesis test results.

Hypothesis	Path	Path coefficient (β)	*P* value	Outcome
Hypothesis 1	Task characteristics to task-technology fit	0.168	<.001	Supported
Hypothesis 2	Technology characteristics to task-technology fit	0.749	<.001	Supported
Hypothesis 3	Task-technology fit to performance expectations	0.508	<.001	Supported
Hypothesis 4	Task-technology fit to perceived risk	–0.281	<.001	Supported
Hypothesis 5	Task-technology fit to perceived ease of use	0.692	<.001	Supported
Hypothesis 6	Perceived ease of use to perceived risk	–0.377	<.001	Supported
Hypothesis 7	Perceived risk to performance expectations	–0.308	<.001	Supported
Hypothesis 8	Performance expectations to intention to use	0.228	<.001	Supported
Hypothesis 9	Perceived ease of use to intention to use	0.108	.07	Rejected
Hypothesis 10	Perceived risk to intention to use	–0.579	<.001	Supported

### Qualitative Data Analysis

From the 48 responses regarding clinicians’ expectations that CDSS fail to meet, 3 themes emerged. The first theme, mentioned 28 times, revolved around reducing system security risks. Clinicians expect a CDSS not only to offer accurate predictions but also to provide transparent explanations for its decisions. This transparency is crucial for fostering trust among health care professionals, ensuring regulatory compliance, and safeguarding patient safety. The second theme, mentioned 10 times, pertained to personalized interactions. Clinicians expressed a desire for a CDSS to move beyond standardized interactions and instead offer personalized conversations. They sought tailored content and forms of interaction that would better meet their individual needs and preferences. The third theme, mentioned 10 times, was related to effective utilization. Clinicians emphasized the importance of efficiently using a CDSS within their busy clinical workflows. Given that time is a scarce resource in health care settings, clinicians expect a CDSS to be designed in a way that seamlessly integrates into their workflows and enhances efficiency rather than adding burdensome tasks.

## Discussion

### Principal Findings and Comparison With Prior Work

As CDSS gain widespread adoption in health care, significant questions arise concerning how they shape performance expectations and perceived risks, as well as clinicians’ willingness to adopt and seamlessly integrate this technology into their clinical workflows. This study marks a pioneering effort at combining the perceived risk theory with the TTF framework, examining how perceived ease of use and the task-technology fit influence clinicians’ perceived risk and performance expectations, thereby impacting clinicians’ willingness to adopt AI systems in their practice.

In our study, clinicians’ willingness to adopt a CDSS varied from moderate to moderately high. We pinpointed several crucial factors that significantly influenced their intention to utilize this technology. Notably, we discovered that perceived risk had a negative impact on clinicians’ intention to use CDSS, with a significant portion of them exhibiting a low level of perceived risk associated with the system. Indeed, perceived risk arises from the system’s lack of transparency. The absence of transparency in a CDSS refers to a deficiency in clarity or openness in how the system makes decisions or generates recommendations. This opacity can foster uncertainty among clinicians regarding the rationale behind the system’s outputs, thereby undermining their trust and confidence in its reliability, hindering their ability to effectively integrate the CDSS into clinical decision-making processes [39]. This finding is consistent with prior research on users’ adoption of mobile service systems, indicating that higher perceived risks associated with new technology use correspond to lower levels of willingness to use it [40]. Additionally, we observed that the perceived risk served as a pivotal mediating factor in the interplay between the task-technology fit and clinicians’ intention to utilize a CDSS. This is due to clinicians’ considerations of the system’s potential risks and uncertainties when evaluating the task-technology fit. When clinicians perceive a low fit between tasks and technology, they may be apprehensive that the system may not adequately support their work demands, subsequently enhancing their perception of risk associated with using the system, ultimately diminishing their usage behavior [41].

We also found that clinicians’ performance expectations for CDSS were at a high level. Our findings indicate a significant positive influence of performance expectations on clinicians’ intention to use a CDSS. This suggests that clinicians are more likely to adopt the technology if they believe it enhances their productivity and contributes to better clinical outcomes for their patients. This is consistent with the findings of a 2021 study that explored the impact of performance expectations on the adoption of AI [42]. At present, the primary factor affecting clinicians’ performance expectations of a CDSS is the system’s inability to effectively integrate into their daily workflows. The main reason is that clinicians have already established a relatively smooth workflow in their daily practice. They are accustomed to using tools and processes that may differ from a CDSS. If the CDSS cannot seamlessly integrate with clinicians’ existing workflows, they may perceive its use as adding to their workload, reducing efficiency, or even causing workflow interruptions [43,44].

Our findings revealed that the accuracy of a CDSS serves as a pivotal determinant of physicians’ adoption intentions. System accuracy not only directly impacts perceived technical utility but also amplifies risk perception among clinicians. It not only directly affects the perceived usefulness of the technology but also significantly heightens clinicians’ perceptions of risk. In practice, physicians’ doubts about a CDSS, especially the risk of systematic errors like diagnostic bias, can erode their trust. In the field of geriatric emergency medicine, for instance, the complexity of clinical decisions, which involves managing multiple diseases and age-related diagnostic bias, can make doctors more aware of CDSS errors. As a result, they may rely more on their experience than algorithm suggestions. Although AI advances may boost CDSS diagnostic accuracy, it is still just a clinical aid, not a replacement. The MDCalc platform, with over 500 evidence-based tools for risk stratification and drug dose calculations, is a case in point. It is designed to enhance, not replace, clinical reasoning. In breast cancer treatment decisions, physicians balance evidence-based medicine and patient-centered values. This shows the irreplaceability of human decisions and the need for human-machine collaboration in complex medical scenarios. Previous studies have shown that an AI-based CDSS had a diagnostic accuracy of 93.6% and a recall rate of 66.5% in 1850 cardiology cases. The study noted that the high accuracy rate enabled physicians to focus on diagnoses quickly, reducing missed diagnoses and delays, thus enhancing their trust in the system and willingness to adopt it. In addition, the system further improved the completeness and efficiency of clinical decision-making by alerting physicians to diagnoses they may have missed [45].

The clinical value of a CDSS hinges on its accuracy, yet medical decision-making inherently intertwines scientific rigor with humanistic considerations. In the short term, a CDSS is best positioned as an intelligent clinical adjunct, mitigating human errors to elevate overall care quality. However, fully supplanting physician judgment remains untenable, constrained by both technological immaturity and the irreplaceable role of human empathy in medicine. Future innovations must prioritize the development of trustworthy, transparent, and interoperable systems that seamlessly integrate into clinicians’ workflows, fostering collaborative human-AI synergy rather than competition.

It is intriguing that, in this particular study, perceived ease of use emerged as insignificant within the context of a CDSS. This suggests that other factors might have played a more dominant role in influencing clinicians’ intentions to use these systems. However, the clinicians’ intentions to use a CDSS were indirectly influenced by the perceived ease of use, mediated through the variable of perceived risk. It is not uncommon to find studies within the realm of information system use where the relationship between perceived ease of use and use intention is deemed insignificant [46,47]. This result underscores that, even if a system is user-friendly, if it fails to deliver tangible benefits in terms of patient care or diagnostic accuracy, clinicians may not be motivated to use it.

Through qualitative data analysis, we pinpointed 3 key areas where clinicians perceived shortcomings in a CDSS: lack of transparency, limited personalized interactions, and inadequate integration with clinical workflows. This revelation provides hospital administrators and system developers with valuable insights into the underlying reasons for the low utilization rates of CDSS. When clinicians encounter a CDSS with opaque algorithms, their perceived risk increases. Additionally, the absence of personalized interactions and seamless integration into workflows diminishes clinicians’ performance expectations, thereby leading to reluctance for continued CDSS usage.

### Managerial and Public Health Implications

Drawing upon the unique characteristics and requirements of clinical tasks, a CDSS can be tailored and optimized to harmonize with clinicians’ operational routines and bolster their decision-making processes. Concurrently, a real-time feedback loop should be embedded within a CDSS to systematically gather clinicians’ ongoing usage feedback and recommendations. This feedback loop facilitates a deep understanding of clinicians’ satisfaction levels and identifies areas for potential improvement. All the aforementioned measures help ensure that the CDSS remains tightly synchronized with the changing tasks and needs of clinicians.

The lack of transparency, interpretability, regulatory and ethical compliance, and accountability issues surrounding AI’s participation in medical decision-making pose a series of challenges in the health care industry, which has sparked the demand for explainable AI in the medical field. Explainable AI not only provides accurate predictions but also offers transparent explanations for its decisions, which is crucial for building trust with clinicians, validating generated insights, ensuring regulatory compliance, and ensuring patient safety [48]. Improving the transparency and explainability of CDSS hinges on integrating technical design with practical application strategies, enabling clinicians to comprehensively understand the system’s decision logic, verify its scientific basis, and build trust. This enhancement should focus on 3 core approaches. First, the “Chain of Diagnosis” framework enables interpretable model design and visualized reasoning pathways by breaking down complex medical diagnoses into clear, sequential steps: symptom abstraction, disease prediction, and confidence assessment. For instance, a dental pain differential diagnosis system uses a symptom-disease mapping matrix to differentiate similar conditions like pulpitis and dental caries. It triggers additional symptom collection commands based on preset confidence thresholds, such as cold sensitivity tests. Using Shapley Additive Explanations, the system quantifies the contribution of key indicators (eg, lactate levels in sepsis prediction) and presents the basis of decision-making through heat map gradients [49]. Second, constructing a dynamic medical knowledge graph based on authoritative guidelines directly links each recommendation to its original evidence source. Clinicians can trace the basis of recommendations through an interactive interface, which includes literature DOI codes, guideline update versions, and evidence level labels. Publicly maintaining knowledge base version iteration logs further ensures system transparency, effectively alleviating trust crises caused by “black box” decision-making [50]. Third, dynamic interaction and multiround consultation designs, based on the information entropy reduction principle, optimized diagnostic workflows. Multiround consultation design reduces diagnostic uncertainty. When system confidence is below a critical value, it automatically starts targeted symptom checks (eg, confirming visual blurring) and shows the impact of new symptoms on differential diagnoses via evolving probability distribution charts. For example, in the DiagnosisGPT system, the real-time updated probability distribution chart visually presents the evolution of hypotheses: The probability of influenza diagnosis drops from 0.4 to 0.1, while that of tuberculosis rises to 0.7. This dynamic reasoning process aligns with clinical thinking, significantly reducing the perceived risk of “arbitrary machine decisions.” Ultimately, a closed-loop framework of “technical verifiability, evidence traceability, and decision controllability” fosters human-AI trust collaboration, ensuring the CDSS aligns with clinical workflows, adheres to ethical standards, and prioritizes patient safety. This triad of strategies bridges the gap between algorithmic outputs and clinical interpretability, empowering physicians to critically evaluate and responsibly act on AI-generated insights.

Although this study did not prove that perceived ease of use can directly increase clinicians’ willingness to use a CDSS, a pleasant system interface, simple and easy operation process, and easy-to-understand information prompts can effectively reduce clinicians’ perceived risk, increase clinicians’ performance expectations, and thus indirectly affect clinicians’ use of the system. Therefore, providing comprehensive user support, including detailed user manuals, online help documents, and video tutorials, ensures that clinicians can easily obtain the necessary help during use, ultimately improving effectiveness and utilization in clinical decision-making.

### Contribution

The contribution of this study is the identification of several key factors that influence clinicians’ use of CDSS. There remains a notable gap, with only a limited number of studies integrating both TAM and the TTF model to comprehensively understand CDSS adoption factors [51,52]. Our study captures the perception of clinicians and degree of technical fit with the task. This study offers a dual contribution. Theoretically, it identifies pivotal factors influencing clinicians’ readiness to embrace CDSS and verifies the model’s applicability and utility via a cross-sectional survey. Practically, the study’s findings furnish tailored managerial recommendations to foster the implementation and efficacy of CDSS, thus bridging the gap between theory and practice in health care settings.

### Limitations

There are some limitations of this study that must be acknowledged. One limitation of the study is the reliance on intention to use as a final variable. Although willingness to use can predict usage behavior, it is important to note that it is not synonymous with actual usage behavior. The study may not fully capture the complex dynamics that affect CDSS utilization in real-world clinical settings. Another limitation of the study is that this study was conducted in 3 tertiary hospitals in Shanghai; extrapolating the results to hospitals with different contextual factors should be done cautiously. Last, the cross-sectional nature of the study may restrict the ability to establish causality between the identified factors and clinicians’ willingness to use a CDSS. Longitudinal studies tracking changes in attitudes and behaviors over time would provide stronger evidence of causal relationships.

### Conclusions

In conclusion, this study set out to uncover the critical factors shaping clinicians’ intentions to use a CDSS. Performance expectations and perceived risk emerged as significant predictors of usage intention. Task-technology fit and perceived ease of use can significantly influence users’ perceived risk and performance expectations. Therefore, CDSS developers must emphasize the advantages of AI technology, align technology objectives with organizational missions (task-technology fit), prioritize a user-friendly design to reduce effort expectancy (perceived ease of use), articulate the system’s capabilities clearly (performance expectancy), and mitigate risk perceptions by refining the overall design. In the future, management policies should encourage the active involvement of clinicians and all stakeholders in the decision-making process concerning CDSS. This participatory approach ensures that diverse perspectives are considered, leading to greater acceptance and buy-in from health care professionals. Furthermore, establishing clear accountability and responsibility frameworks can foster trust and confidence among users, guiding the use of AI technology. By implementing these measures, organizations can mitigate risk perception, enhance performance, and ultimately increase clinicians’ intentions to integrate CDSS into their daily practice.

## Appendix

Multimedia Appendix 1 Operationalization of the research variables.

## Abbreviations

AI: artificial intelligence
AVE: average variance extracted
CDSS: clinical decision support system
SEM: structural equation modeling
TAM: technology acceptance model
TTF: task-technology fit
