# Supplementary material for: Investigating Clinicians’ Intentions and Influencing Factors for Using an Intelligence-Enabled Diagnostic Clinical Decision Support System in Health Care Systems: Cross-Sectional Survey
Source: J Med Internet Res. 2025 Apr 7;27:e62732. doi: 10.2196/62732 (PMC12012391; doi:10.2196/62732)
Supplement: Multimedia Appendix 1 [file jmir_v27i1e62732_app1.docx]

**Appendix 1**

Operationalization of the research variables.

| **Construct** |  | **Measurement items** | **Source** |
| --- | --- | --- | --- |
| Task Characteristics |  |  |  |
|  | TAC1 | In my job, I need to apply a significant amount of medical knowledge or data. | Goodhue |
|  | TAC2 | In my job, I require robust evidence to support my decisions |  |
| Technology Characteristics |  |  |  |
|  | TEC1 | CDSS can generate accurate and reliable diagnostic results | Goodhue |
|  | TEC2 | CDSS can recommend effective treatment plans |  |
| Task-Technology Fit |  |  |  |
|  | TTF1 | Based on CDSS's performance in assisting me with tasks, its functionality is useful | Wells |
|  | TTF2 | Based on CDSS's performance in assisting me with tasks, the functionality of CDSS can support my work |  |
| Performance Expectations |  |  |  |
|  | PE1 | CDSS does not increase the difficulty of our daily work | Venkatesh |
|  | PE2 | CDSS can enhance my work efficiency |  |
|  | PE3 | CDSS can improve my level of diagnosis and treatment |  |
| Perceived Ease of Use |  |  |  |
|  | PEOU1 | I find CDSS easy to operate and control | Davis |
|  | PEOU2 | "I find the interface of CDSS pleasant and satisfying |  |
|  | PEOU3 | I find the CDSS information prompts concise, time-saving, and minimally intrusive |  |
|  | PEOU4 | I find the content and presentation of information provided by CDSS prompts easy to understand |  |
| Perceived Risk |  |  |  |
|  | PR1 | I believe that the results from CDSS could lead to erroneous judgments | Stone |
|  | PR2 | I believe that using CDSS could waste a significant amount of my work time |  |
|  | PR3 | I believe that CDSS could increase my workload burden |  |
| Intention to Use |  |  |  |
|  | ITU1 | I will use CDSS in my work | Venkatesh |
|  | ITU2 | I will use CDSS to help complete more clinical tasks |  |
